# Various epileptic seizure detection techniques using biomedical signals: a review

**DOI:** 10.1186/s40708-018-0084-z

**Published:** 2018-07-10

**Authors:** Yash Paul

**Affiliations:** School of Informatics, Eötvös Loránd University, Budapest, Hungary

**Keywords:** Fourier transform, Wavelet, Epilepsy, Electroencephalogram (EEG), Hilbert transform, Empirical mode decomposition, Rational function, Particle swarm optimization (PSO)

## Abstract

Epilepsy is a chronic chaos of the central nervous system that influences individual’s daily life by putting it at risk due to repeated seizures. Epilepsy affects more than 2% people worldwide of which developing countries are affected worse. A seizure is a transient irregularity in the brain’s electrical activity that produces disturbing physical symptoms such as a lapse in attention and memory, a sensory illusion, etc. Approximately one out of every three patients have frequent seizures, despite treatment with multiple anti-epileptic drugs. According to a survey, population aged 65 or above in European Union is predicted to rise from 16.4% (2004) to 29.9% (2050) and also this tremendous increase in aged population is also predicted for other countries by 2050. In this paper, seizure detection techniques are classified as time, frequency, wavelet (time–frequency), empirical mode decomposition and rational function techniques. The aim of this review paper is to present state-of-the-art methods and ideas that will lead to valid future research direction in the field of seizure detection.

## Introduction

**Fig. 1:**
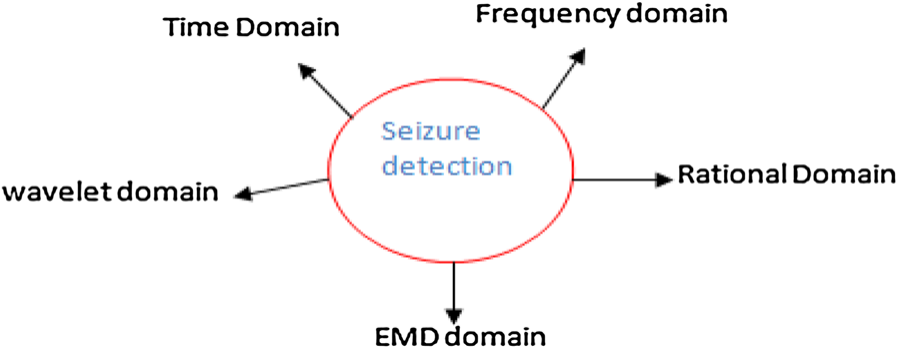
Classification of seizure detection method

Epilepsy is a neurological disorder which creates severe effects to human brain. According to the latest study, more than 2% of the population worldwide is affected from epilepsy where 85% of those live in developing countries and has adverse effects on their daily life and productivity. Each year 2.4 million new cases are estimated to occur globally [1, 2]. EEG signals are usually used by experts for the diagnosis of the epilepsy. EEG signals are classified into two types: (a) scalp EEG and (b) intracranial EEG (iEEG). Scalp EEG is captured by placing the electrodes on the surface of scalp by using international standard 10–20 system [3]. iEEG signals are captured by placing the electrodes directly on the surface of brain to record the brain activity from the cerebral cortex. Detecting and locating the seizure period in EEG recordings manually is difficult and time-consuming because EEG recordings are usually tens or even hundreds of hours long. Epilepsy seizures are mainly classified as *generalized or local* and *partial seizures* or focal. Generalized seizures are further classified as Grand Mal, Absence, Myoclonic, Clonic, Tonic and Atonic and are produced by electrical impulses throughout the whole brain, whereas partial seizures are categorized as simple and complex and are from small portion of the brain. Simple seizures are simple motor, simple sensory and simple psychological, and complex seizures are partial seizure with secondary generalization. In seizure signal, four states are identified, namely pre-ictal, ictal, inter-ictal and post-ictal. The portions of the signal before the first seizure and after the last are called pre-ictal and post-ictal. Ictal and inter-ictal indicate intervals of seizures and between seizures. When a seizure occurs, it might cause injuries or jeopardize the life of the patients especially when they are driving cars or working with different machinery. That is why there is a need to develop an automatic seizure detector to avoid different types of harms to epileptic patients. Most of the research work is carried out by using scalp EEG, because capturing the signal from the surface of the brain (iEEG) is quite risky and require lots of expertise in it. There are number of review papers published on this area, but most of the papers do not cover the complete state-of-the-art methods and transforms like application of rational transform in seizure detection. According to [4, 5], the seizure detection process is classified as single-channel or multichannel process. In single-channel process, a channel or signal which is strong and close to the seizure origin is selected based on some measures like *local variance.* Combining the information from more than one channel through some data fusion techniques [6] gives better results in seizure detection process. Another attempt to classify seizure detection as *linear* and *nonlinear* techniques is made in [7–9]. Tzallas et al. [10] classified seizure detection methods as pattern recognition methods, morphological analysis methods, parametric methods, decomposition methods, clustering methods and data mining methods. Alotaiby et al. [11] classified seizure detection methods based on time, frequency wavelet and empirical mode decomposition (EMD) domains. We adopt classification based on [11] but with latest methods and additional domain called rational domain and discuss how rational transform is more efficient than wavelet transform and other methods while detecting seizures from a given EEG epochs (time window). Because of the application of various transforms like discrete Fourier transform (DFT), discrete wavelet transform (DWT), Hilbert transform, Gabor transform, rational transform, etc., decomposition techniques like empirical mode decomposition, singular value decomposition, etc., and data reduction techniques like principal component analysis (PCA) and independent component analysis (ICA) have played an important role in seizure detection. In this paper, we are reviewing the latest directions in seizure detection area and the techniques and methodologies used are categorized into five domains, i.e. time domain, frequency (DFT) domain, wavelet domain (time–frequency), empirical mode decomposition (EMD) and rational transform domain. Most of the techniques discussed here are noninvasive. Based on the various processing domains, the classification of the various seizure detection methods is shown in Fig. 1.

In this study, we also summarize some papers which adopted other biomedical signals like electrocardiogram (ECG), electrocorticography (ECoG), etc., or combination of different signals for seizure detection. We focused on scalp EEG databases-based techniques and methodologies.

The performance of the most of the techniques in different domains is measured by following quantities:

$${\text{Sensitivity}} = \frac{\text{TP}}{{ {\text{TP}} + {\text{FN}}}} \times 100$$

$${\text{Specificity}} = \frac{\text{TN}}{{{\text{TN}} + {\text{FP}}}} \times 100$$

$${\text{Accuracy}} = \frac{{{\text{TP}} + {\text{TN}}}}{{\begin{array}{*{20}c} {{\text{TN}} + {\text{FP}} + {\text{TP}} + {\text{FN}}} \\ \end{array} }} \times 100$$

where TP → true positive, is the number of epochs which are marked as seizure by both algorithm and doctor.

TN → true negative, is the number of epochs which are marked as non-seizure by both algorithm and doctor.

FN → false negative, the number of seizure epochs which are misclassified by the algorithm, i.e. recognized as non-seizure but actually they are seizures.

FP → false positive, the number of non-seizures epochs which are misclassified by the algorithm, i.e. recognized as seizure but actually they are non-seizures.

The performance of the seizure detection algorithm primarily depends on following parameters:

Transformation technique, feature selection, classifier used, window size, type of window or mother wavelet, the levels of decomposition of the original signal and optimization algorithm, etc.

We will also investigate above-mentioned parameters from different domains, and it will help the researchers to identify which parameters are more relevant to their algorithms for further improvement in the already-existing seizure detection methods. The study will also help the readers to become familiar with different types of public and non-public epilepsy databases.

The paper is organized as follows. It has four sections. Section 1 is related to time and frequency domain methods, Section 2 is about wavelet domain, in Section 3, EMD domain is discussed, Section 4 contains rational transform domain and comparisons of various techniques in terms of feature, methods and results. Every section presents the core idea of the papers along with what features are used, which transform is used, what is the appropriate window size, what is the signal duration, what type of results, i.e.  % of sensitivity, specificity, accuracy false detection rate, etc. The 4th section also includes the conclusion, various problems and valid directions of future research in this area.

## Section 1

### Time domain or threshold-based methods

Time domain refers to how the value of the signals varies over time, in other words time parameter is the independent variable of the signal. Time domain methods are usually patient-specific or problem-specific and do discrete time analysis, and do analysis of the given epochs (time window). Thus, this is value-time analysis of a given signal *x*(*t*). Seven papers are summarized here. The main objective here is to demonstrate the various approaches, interconnection among approaches and different possibilities in time domain so that we can further do improvement in seizure detection devices. To this end, we selected seven different papers with different ideas. The selected papers have high accuracy, sensitivity, specificity and low false detection rate. It may be beneficial to the researchers who are interested to develop seizure detection devices with high speed and more accuracies. The performance in terms of accuracy, sensitivity, specificity and false detection rate of below mentioned algorithms depends mainly on core ideas, selection of the features and classifiers being used. Since these methods do not require transformations and are generally fast and are used in seizure detection devices like smart watch, tablets, etc., proposed algorithms are tested on CHB-MIT, Bonn database [12] and self-recorded data, whereas last two papers are purely hardware based.

Shanir and Khan [13] Proposed method for automatic seizure detection based on mean and minimum value of energy per epoch, i.e. mean of the energy of each sample point in a epoch and sample point having minimum energy in a epoch are used as features for classification. The window size was chosen as 1 s. The classifier used here is linear classifier. The algorithm was tested on CHB-MIT database on three subjects with 60 and 40% of data used as training and test data, respectively. They obtained an average detection accuracy of 99.81%, sensitivity 100%, and specificity 99.81%. The epoch having seizure, the features like mean and minimum of energy have greater values than the segments with no seizure and can be classified easily as seizure and non-seizure epoch. Features are extracted here from original epoch, and therefore, the size of the feature vector is large which may reduce the speed of the system. This large size vector may be reduced by half-wave and histogram method [2, 14].

Alotaiby et al. [2] They proposed patient-specific method for channel selection and seizure detection by estimating the histograms of multichannel scalp EEG signals. In the training phase, the signal is windowed by using 10-s-long non-overlapping window with five histograms measured from each segment. Histograms have number of bins that are studied individually as random variables. Based on some predefined threshold, bin(s) are selected from certain channel distributions for seizure detection. During training hours, a leave-one-out cross-validation technique is applied. In the testing phase, those channel(s)-histogram(s)-bin(s) are used to classify each segment as ictal or non-ictal. The resulting sequence is de-noised with a moving average filter and compared to a patient-specific detection threshold. The method is tested on CHB-MIT dataset using 309.9 h of sEEG including 26 seizures of five patients. They have shown an average sensitivity and specificity off 97.14% of 98.58%, respectively.

Runarsson and Sigurdsson [14] The idea behind this paper is: first, find the half-wave form of the EEG epoch at hand and then find the consecutive peaks and minima in that half-wave signal segment. The histograms are estimated for two variables: the *amplitude difference* (*Δ, Y*-axis) and *time separation* (*τ, X*-axis) between two consecutive peak values as well as minima. Here we have two histograms one for minima and other for maxima. The features used for classification of an epoch as a seizure or non-seizure are these estimated values like *Δ and τ* from local minima and maxima. Actual features used are the frequencies of co- occurrences of *τ* and *Δ* and each feature is generated from 8 s long signal with 2 s overlap 12 h self-recorded data using 10 EEG channels with 256 sampling. Support vector machines (SVMs) with chunking method are used as classifiers. An average sensitivity of about 90% is achieved. Proposed algorithm in [14] may not perform better than [13] on CHB-MIT because CHB-MIT has very long duration data and requires investigation. On the other hand most of the values in the feature vector extracted by half-wave method will be zero and it gives the sparse representation of the data and hence less data to be processed as compared to the methods in [2, 13]. Problem with this method is that actual processing of the signal like extraction of the features starts only after finding the half-wave representation of the original signal. Once the half-wave is in hand this could be very fast algorithm in time domain applications with large amount of dataset and can be used as online seizure detection method. In [15] the researchers designed the seizure detector (hardware) and implemented the algorithm (software) in the designed processor. In [16] they have developed a improved network of seizure detection devices.

Mursalin et al. [17] Present a hybrid approach where features from time and frequency domains are analysed to detect epileptic seizure from EEG signal. Time domain features like mean, median, mode, minimum, maximum, skewness, standard deviation, kurtosis, first quartile (Q1), third quartile (Q3) and interquartile range (Qir), mobility and complexity, Hurst exponent and the detrended fluctuation analysis with frequency domain features like Maximum of the wavelet coefficients, Minimum of the wavelet coefficients Mean of the wavelet coefficients Standard deviation of the wavelet coefficient. First they apply improved correlation-based feature selection (ICFS) method to select prominent features from the time domain, frequency domain, and entropy based features and these then classification of the selected feature is done by an ensemble of Random Forest (RF) classifiers. Results show that the proposed method is better in performance as compared to the conventional Correlation-based and some other state-of-the-art methods of epileptic seizure detection methods when tested on Bonn dataset.

Alejandro and Ramon-Lozano [18] In this paper, authors used energy of the signal in a different way. They used smaller window as the foreground windows and larger window as the background while windowing the signal. Energy is calculated in every foreground and background window, and the energy ratio is calculated by dividing the foreground energy by the corresponding background window energy resulting a series of energy ratios and can be treated as a time series distribution where some values are more higher than average or threshold values are part of seizure. Finally after thresholding, the values are combined and seizures that are shorter than l_min_ (the minimum seizure duration.) seconds are deleted which may removes noise too. Finally, the algorithm has detected a set of seizures for each channel. The final set of detected seizures is a result of all different channels. All above-mentioned steps for seizure detection require some set of optimized parameters, and these parameters are optimized by genetic algorithms. The algorithm is tested on CHB-MIT database and found that the number of false positives is very small, and it is 0.39 per 24 h in average, which less than most state-of-the-art methods.

Yoo et al. [15] They designed a multichannel-based processor called system on chip (SoC) for detecting the seizure, and energy of the signal is used as features. The feature vector is constructed by dividing the signal into segments, and instead of calculating the energy of the signal as whole they calculated the energy from these segments, which results in a more discriminative feature vector. The device has 8 data acquisition channels, feature extraction module (FE) and classification engine (CE). They used SVM as classifier and is trained to detect rapid-eye blink patterns as this is similar to the generalized seizure and has more energy as compared to non-seizure patterns. The SoC was tested on CHB-MIT scalp EEG database [19] and it showed an accuracy of 84.4% with a total time of 2 s and 2.03 μJ/classification energy. The advantage of this processor as compared to IAS processor is that it enlarges the EDO filtering range up to + 200 mV which is 4 times better and consuming the same power of 2.5 μW.

Dalton et al. [16] In [15], the processor has the capability to save power, by working in a single mode at a time, keeping other mode off at the same time, but device is not used in network, i.e. it is standalone device for seizure detection. Dalton et al. proposed the algorithm which can be used in standalone device as well as in the network too. It is a single channel method where they developed a body senor network (BSN) that can monitor and detect epileptic seizures using the features like mean, variance, zero-crossing rate, entropy, root means square (RMS), and autocorrelation with template signals extracted from time domain signals. They detected motor seizures, and data are collected by using accelerometer-based kinematic sensors like gyroscope and magnetic sensor for physical activity monitoring. They adopted a dynamic time warping (DTW) approach for best alignment between the signal segment to be tested and the template signal, i.e. no classifier is used for classification. They also found that RMS is best separation feature as compared to others listed above. The sensitivity and the specificity of the algorithm for a dataset of five subjects with 21 seizures is 91 and 84%, respectively, with battery lifetime of 10.5 h. The problem with this technique is that they used N810 tablet, and it does not contain cell phone capabilities. The device is GPS-enabled and can send only messages to another cell phone or device. Therefore, we can conclude that time domain methods are simple and are good choices to implement these algorithms for patient-specific problems, seizure detection devices and in networks too. The methods proposed in [2, 13, 14, 17] may further improve the performance of the seizure detection devices because of their high accuracy and sensitivity.

*Observations* The problem with time domain analysis is that it does not tell about the frequency components of the signal and this drawback is overcome by frequency domain analysis which provides deeper analysis of the signal as compared to time domain. Time domain methods are usually fast and can be used in real-time systems.

### Frequency domain

Time domain method does the analysis of the signal based only the time and magnitude components of signal [magnitude (*Y*-axis), time (*X*-axis)], and there is no information about frequency component of the signal. But if we want the deep analyses of the signal then frequency component is also required. Frequency domain tells about the frequency spectrum [magnitude (*Y*-axis), frequency (*X*-axis)] of the signal. The advantage of the transformation of signal from one domain to another domain is that it provides insight and points out the important properties of the signals which cannot be seen by visual inspection of the original signal and or hidden signal in time domain. Theoretically, signals are decomposed into pure sinusoidal signals with different frequencies. The signal is transformed to frequency domain by using Fourier transform, and Fourier transform’s magnitude and phase can be exploited here. Thus, this is amplitude–frequency analysis of the given signal. Frequency domain methods are more robust than time domain methods. Five papers are discussed to demonstrate unique ideas for seizure detection. The proposed algorithms perform excellent on long-term recordings and help to distinguish different states of seizure EEG signal. This domain like time domain methods also helps us to select a channel having greater seizure activity.

Bhople [20] Authors proposed epileptic seizure detection method by using fast Fourier transform (FFT). The FFT-based features are extracted and are fed to the neural networks. They used multilayer perceptron (MLP) and generalized feed-forward neural network (GFFNN) as a classifier. The algorithm is tested on Bonn database, and results show they are able to achieve 100% accuracy.

Hills [21] The author participated in a competition “UPenn and Mayo Clinic’s Seizure Detection Challenge” and he used fast Fourier transform (FFT) to each one second long window and taking magnitude in the range 1–47 Hz and leaving phase information. Then correlation coefficients and eigenvalues are computed in both frequency and time domains and added to the FFT data to form the feature vector; these features are classified using random forest classifier with 3000 trees.

Rana et al. [19] They proposed a multichannel algorithm for seizure detection, and their algorithm is based on phase slope index (PSI). PSI metric of EEG signals is used to categorize seizure and normal activities. The PSI metric measures the interaction between the two channels and identifies rise in the spatio-temporal interactions between the channels, which undoubtedly differentiate seizure from inter-ictal activity. It is a threshold-based method where threshold is chosen by using moving average of latest activity to include differences between patients and slow changes within each patient over time. The performance of the algorithm is tested on 258-h-long recorded EEG data of five patients with different types of epilepsy. ECoG data of five patients ranges from 41 to 63-h-long and have 5–15 seizures in each case. The performance of the algorithm was tested for two segments of 20 and 60 s long without using any classifier, and they showed that the algorithm successfully detected all seizures from all 5 patients and achieved false detection rate lower than two per hour. The 20-s-long segment and a threshold of 4.8 standard deviations detected 100% of the seizures excepting in one patient. A unique strength of this paper is that it is designed and evaluated on long-term recordings. They also showed that their algorithm can be used to find the channels among various channels having strong activity.

Khamis et al. [22] It is a single channel, patient-specific and with no threshold parameter method of seizure detection. Frequency domain features like frequency moment signatures are used to distinguish a seizure segment from non-seizure. The EEG signals at hand have marked the collected scalp EEG data with seizure events. After marking the signal, a filtering process has been performed on the windowed EEG data from electrode differences T6-P4 (right hemisphere, RH) and T5-P3 (left hemi sphere, RH). *Power spectral densities* of the signals on both hemispheres have been computed and frequency components of both hemispheres are combined into a single quantity. Signatures are calculated by subtracting normalized central moments of the subset distribution from the mean, and it shows whether the amplitude of the seizure frequency is consistent and it helps to distinguish a seizure from a transient event. The blocks (RH blocks and LH blocks) of 32 s from 8192 are merged with 50% overlap, resulting in a signature in every 16 s. A triangular window having 1024 sample size with 50% overlap is used resulting in a total of 15 subset transforms from each RH and LH blocks. Any frequency which has consistent spectral power at a give time in all the subsets is seizure frequency and is different from any normal event. They used template matching algorithm called “Powell’s direction set” method for classification, i.e. to measure the consistency of a frequency. A sensitivity of 91% and false alarm rate of 0.02 false positives per hour is achieved as per this method. Thus, no threshold parameter and no classifier are used for classification. The algorithm is tested on 618-h-long recordings.

Acharya et al. [23] They designed a method for the detection of three states of EEG signal, i.e. *normal, pre*-*ictal, and ictal* conditions from recorded EEG signals. They combine the features from two domains, i.e. time domain and frequency domain and found that this combined features method is performing good in situations when signal has nonlinear and non-stationary nature. Four entropies (measure of randomness), phase entropy 1 (S1), phase entropy 2 (S2), approximate entropy (ApEn), and sample entropy (SampEn) are used as features. The phase entropies are estimated from the higher-order spectra of EEG signal epochs and can be used as discriminating features for ictal, pre-ictal, and inter-ictal activities. The approximate and sample entropies are logarithmic measures that measure the closeness and matching between the incoming EEG signal pattern and the recorded templates. The entropy features are fed to seven different classifiers like SVM, fuzzy Sugeno classifier (FSC), probabilistic neural network (PNN), KNN, naive Baye’s classifier (NBC), decision tree (DT), and Gaussian mixture model (GMM) for comparison, and finally they compared the results. Results showed that Fuzzy classifiers are optimal, with an accuracy of 98.1%.

*Observations* Frequency domain methods are good choices when recorded data is large, i.e. for long-term data but time component of the signal is missing here. On the other hand, combination of features from different domains may produce very promising results. But for more efficient analysis of the signal we need a transform that will tell us about time and frequency components of the signal simultaneously and such type of transformations are discussed in next section.

## Section 2

### Wavelet domain (time–frequency)

A wavelet can be defined as a waveform with certain properties: (a) effectively limited duration and (b) zero average value.

*Example* 
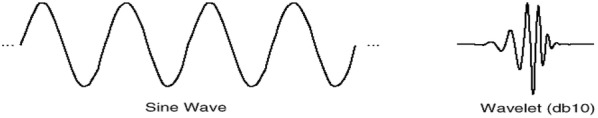


**Fig. 2:**
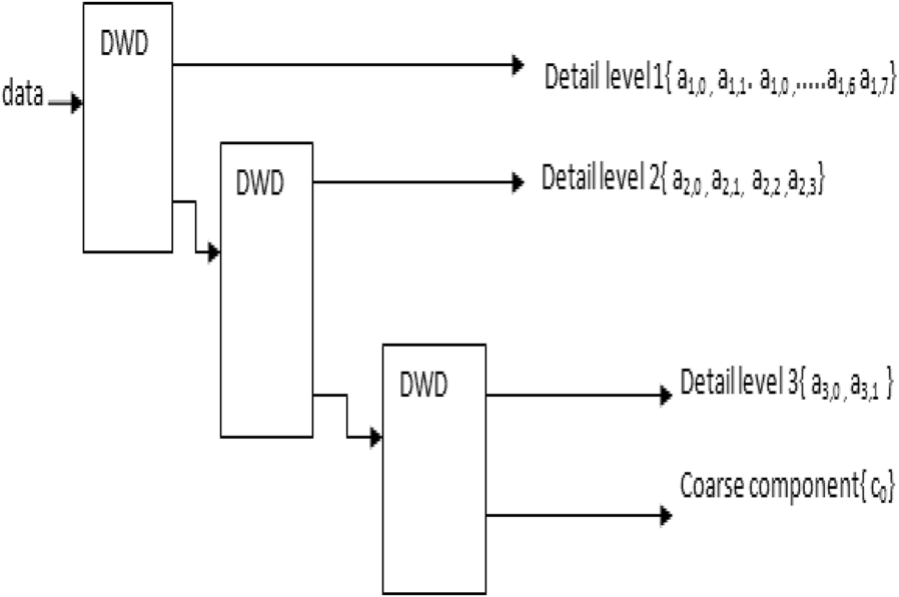
Discrete wavelet decomposition (3 levels)

Here basis functions are wavelets called mother wavelets, e.g. Har, Daubechies, etc. The mother wavelet is a reference wavelet, whose coefficients are evaluated for the entire range of dilation and translation factors [24]. Wavelet transform is a time–frequency analysing tool like short Fourier transform (STFT), which is found very useful to extract the features from the signals which are non-linear and non-stationary in nature like EEG signals. It gives better time and frequency localizations as compared to the short time Fourier transform. It uses long time windows for low-frequency components and short time intervals for high-frequency components. Fourier transform has fixed basis functions throughout signal and generally extract global features of the signals. STFT can be used to extract local feature, but it has some drawbacks like fixed window size and dilemma of Resolution, i.e. narrow window gives poor frequency resolution, while wide window gives poor time resolution. Wavelet transform uses scale-varying basis functions to compute the energy of a signal and extract local features of the signal. Fourier transform holds Heisenberg uncertainty principle, i.e. it cannot know what frequency exists at what time intervals. Wavelet transform decomposes the signal into sub-bands or decomposition levels, and then, features are extracted from the selected sub-bands or levels. The main challenging task in wavelet-based EEG seizure detection is finding the appropriate number of decomposition levels, mother wavelet and the selection of the features from certain sub-bands to discriminate seizure from non-seizure periods. Discrete wavelet decomposition (DWD) provides sufficient information both for analysis and synthesis of the original signal, with a significant reduction in the computation time. DWT signal is also continuous like continuous wavelet transform but scaling and translation parameters here are discrete in nature. Discrete wavelet transform can be implemented with single level or multilevels. Three-level discrete wavelet decomposition (DWD) is shown in Fig. 2.

Wavelet function is defined as follows:

1 $$\varPsi_{s,\tau } (t) = \frac{1}{\sqrt s }\varPsi \left( {\frac{t - \tau }{s}} \right)$$

$\tau \to$ shift parameter and *s* $\to$ scale parameter.

Wavelet transform:

2 $$\gamma \left( {s,\tau } \right) = \int {f\left( t \right)\varPsi_{s,\tau }^{*} \left( t \right){\text{d}}t}$$

Inverse wavelet:

3 $$f\left( t \right) = \iint {\gamma \left( {s,\tau } \right)\varPsi_{s,\tau } \left( t \right){\text{d}}\tau {\text{d}}s}$$

The wavelet coefficients are shown as pixel intensity (colour), in a two-dimensional plane with *y*-axis in lieu of the scaling factor of the wavelet, and the *x*-axis, its translation time axis. The wavelet transform plot, i.e. scalogram is a two-dimensional colour pattern representing the location of the “event” occurring in the timescale.

The main motive of this section is to make the readers familiar with time–frequency analysis using wavelet transform. When we are proposing the algorithms for seizure detection using wavelets, it is necessary to know how many decomposition levels are sufficient for efficient seizure detection, which classifier is appropriate and what types of mother wavelets are good for seizure detection. To this end, we have chosen 10 papers. The algorithm proposed in first three papers are tested on same database, i.e. Bonn database and hint us about what type of mother wavelet is appropriate for efficient seizure detection. Next six papers investigate the appropriate decomposition levels of the signal for efficient seizure detection, and last paper is hardware based. Different classifiers are discussed in all ten papers.

Hasan et al. [25] Proposed a new method for seizure detection by using Wavelet and Hilbert transforms (explained in section 3). The features like Mean, maximum, minimum, standard deviation and average power of absolute values of wavelet and Hilbert transform coefficients are extracted separately. The decomposition level 2 was chosen because dynamics of EEG signals contain important sub-bands. Daubechies wavelet of order 4 (db4) is chosen as mother wavelet. The K-nearest neighbourhood (KNN) classifier is applied separately on these extracted features. The performance is tested on Bonn database [26], and they found that the results obtained by using Hilbert transform are quite promising. Accuracy: for wavelet case, 100 and 96% for the A–E and B–E datasets, and for Hilbert case, 100 and 100% for the A–E and B–E datasets, respectively. In case of Hilbert transform, they have also shown the sensitivity and specificity of 100% each. The power of the algorithm is that it uses only one decomposition level achieving an highest accuracy.

Zainuddin et al. [27] In this paper, the authors first take the wavelet transform of EEG signals to generate a set of coefficients, and then, maximum, minimum, and standard deviation of the absolute values of the wavelet coefficients in each sub-band are extracted as features. The extracted features are then classified by WNNs (wavelet neural networks) classifier. They also investigated various mother wavelets like Gaussian, Mexican Hat, and Morlet, and they found that the best performance was obtained with WNNs using a Morlet wavelet activation function with order 4 Daubechies wavelet for feature extraction. They used **Bonn database** to evaluate the performance of proposed method and achieved sensitivity and specificity up to 98%. Thus, algorithm proposed in [27] with Hilbert transform is much better in terms of sensitivity and specificity than algorithm proposed in [27] on Bonn database and can be used in real-time applications.

Niknazar et al. [28] They used recurrence quantification analysis (RQA) a well-known and well-suited analysis technique for nonlinear data on recorded EEG, and their alpha, beta, delta, theta, and gamma sub-bands are extracted by a four-level Daubechies wavelet transform. The signal is decomposed into five levels. After extracting the features, an error-correcting output coding (ECOG) classifier is used on Bonn database to classify the three states like normal or healthy, inter-ictal, and ictal. They achieved an accuracy of 98.67%.

Therefore, from these three papers we can conclude that authors investigated different mother wavelets to decompose the signal and found that in many cases the results with Daubechies (db2, db4) and Morlet are exceptionally good.

Zhou et al. [29] This paper used lacunarity and fluctuation index as features, and Bayesian linear discriminant analysis (BLDA) is used as classifier.

The lacunarity (gap) is a measure of heterogeneity, and fluctuation Index can measure the intensity of the fluctuation of EEG signal. The ictal EEG epoch has larger fluctuations index and Lacunarity values than the inter-ictal or normal epoch. Authors first decompose the EEG epochs into five decomposition levels (sub-bands). Three sub-bands or levels with scales three, four, and five are chosen for the extraction of features. Features like lacunarity and fluctuation index are computed from these selected frequency sub-bands. The performance of this algorithm is evaluated and investigated on Freiburg EEG database [2], and it is found a sensitivity and false detection rate of 96.25% and 0.13/h, respectively. This algorithm mainly focused on sensitivity and false detection rate, whereas false detection rate is not mentioned in [25, 27, 28].

Chen et al. [30] In this paper, they decompose the EEG signals up to six wavelet scales without down-sampling. Scales 3, 4, 5, and 6 are chosen for further processing. The fast Fourier transform on selected scales has been performed, and magnitude of the Fourier coefficients is chosen as features for seizure detection. Nearest neighbour classifier is used to classify the input EEG signal into the seizure and non-seizure class. The performance of the proposed algorithm is tested on Bonn database and perfect correct classification rates achieved (100%) for all seven binary classification problems, and it is better than existing methods like [25, 27] in terms of classification rate.

Liu et al. [31] It is also a five-level decomposition technique. Three wavelet sub-bands are selected for feature extraction and feature selection. The feature used is relative amplitude, relative energy, coefficient of variation, and fluctuation index from the selected three levels. The fluctuation index is a measure of the intensity of a decomposed sub-band at hand. The classifier used here is SVM, and after classification some kind of post-processing like smoothing, multichannel decision fusion, and collar is done to further enhance the seizure detection performance. The collar processing is a technique that is used to maintain the data continuity during processing. The results are tested on 509 h for 21 epilepsy patients, and they found sensitivity, specificity and false detection rate of 94.46, 95.26%, and 0.58/h, respectively. This algorithm is robust as compared to above-mentioned techniques because algorithm is performing exceptionally well on long-range data.

Abbasi and Esmaeilpour [32] The objective of this paper was improving the precision of prediction and classifying different states of EEG signals into healthy, convulsive, and epileptic states. In this approach, they divide the signal into 5 levels. For further processing, they chose first 4 levels and last, i.e. 5th low-frequency level is rejected. Features like maximum, minimum, average and standard deviation for each sub-band are extracted. A multilayer perceptron (MLP) neural network was used as classifier. The confusion matrix was used to calculate the performance, and the algorithm tested on Bonn database achieved an accuracy, sensitivity and specificity of 98.33, 100, and 97.1%, respectively. Mother wavelet used here is Daubechies-4.

Panda et al. [33] It is also a five-level decomposition technique for features extraction. The extracted features are energy, standard deviation, and entropy. Reference wavelet used here is Daubechies (db-2), and SVM is used as a classifier. They compared the results of individual features and found energy feature has highest accuracy of 91.2%. The algorithm is tested by detecting the seizure activity on 500 epochs of EEG data (100 epochs from each activity) from five different brain activities like eye close, eye open, seizure, hippocampal region, and opposite of epileptogenic zone.

Khan et al. [34] Authors use the same technique as Panda et al. [33], but choose different set of features like relative energy and a normalized coefficient of variation (NCOV). It works on wavelet coefficients acquired in the frequency range of 0–32 Hz. The algorithm is tested on five patients from CHB-MIT scalp EEG database, and they found the performance of NCOV ($${\raise0.7ex\hbox{${\sigma^{2} }$} \!\mathord{\left/ {\vphantom {{\sigma^{2} } {\mu_{a} }}}\right.\kern-0pt} \!\lower0.7ex\hbox{${\mu_{a} }$}}$$) over the traditionally used COV ($$\sigma^{2}$$/$$\mu^{2}$$) is better. Reference wavelet used here is Daubechies (db-4), and they achieved an overall accuracy, sensitivity, specificity, and precession of 83.6, 100, 91.8, and 86.7% respectively. $$\sigma^{2} \,{\text{and}}\, \mu_{a}$$ are variance and mean, respectively, of the epoch. After studying these six papers, we can conclude that five-level decomposition of the signal is sufficient for seizure detection.

Shoaib et al. [35] They develop a processor for seizure detection that directly uses compressively sensed electroencephalograms for embedded signal analysis. Their main aim to present this method is that it saves energy of the processor through compressive sensing. The wavelet energy is used as features. By analysing the compressed signals directly, it avoids reconstruction costs, computational energy of signal analysis due to the reduced number of signal samples. Their results showed that, because of compressive sensing there is 4% decline in sensitivity, 0.15/h increase in false alarm rate, and a latency of 1 s as compared to baseline performance. The results were tested on CHB-MIT database with SVM as classifier. For linear SVMs, the total processor energy lies in the range of 0.3–2.2 μJ, for nonlinear support vector machines energy lies in the range of 12.6–38.5 μJ by using fourth-order polynomial kernel, and 18.1–53.3 μJ for SVMs with an RBF kernel. After every 2 s, classification results are produced, and for all SVMs kernels the total processor power appears in the range 0.6–107 W.

*Observations* Applications of wavelets in signal processing tremendously increased the accuracy in signal processing techniques. We can conclude that decomposition level up to 5 is sufficient for seizure detection. It is difficult to recommend a particular classifier while dealing with wavelets but SVM, artificial neural network and KNN may be good options for classifications. Daubechies wavelet is frequently used, and results are quite interesting.

## Section 3

### Empirical mode decomposition (EMD)

In this section, first we will give introduction about EMD and Hilbert transform since the selected papers here are mainly based on these two techniques. EMD is also a time–frequency analytical technique which is independent from the Fourier and wavelet domains. Fourier and wavelet transforms have prior fixed basis, whereas EMD is adaptive in nature and do not require any prior fixed basis for analysing nonlinear and non-stationary signals like EEG. It is a nonlinear data decomposition technique, which is developed by Huang et al. [36]. In signal processing, EMD is a signal decomposition technique, in which a signal is transformed into a group of functions or components called intrinsic mode functions (IMFs), which preserve the inherent properties of the original signal. As the decomposition level increases, the complexity of the IMFs decreases, and so does the scale of the signal. When there are abnormal activities in brain signals, these IMFs show different behaviour than normal activities. Therefore, various features can also be extracted from the IMFs and even IMFs itself can be used as features for seizure detection. EMD algorithm is given below:

Given a signal *x*(*t*),

1. Extract the maxima and minima points of the signal and
2. interpolate between them to determine the maximum and minim envelopes.
3. Using these envelopes we calculate the local mean, *m*(*t*) as: $m\left( t \right) = \left( {e_{\hbox{min} } \left( t \right) + e_{\hbox{max} } \left( t \right)} \right)/2$ here *e*_min_ and *e*_max_ denotes the minimum and maximum envelope
4. Extract the detail *d*(*t*) = *x*(*t*) − *m*(*t*)
5. If *d*(*t*) does not match the criteria of an IMF then the procedure is iterated at step 1 with the new input *d*(*t*) and we skip steps 6 & 7
6. If *h*(*t*) matches the criteria of an IMF, it is stored as an IMF, $g_{i} \left( t \right) = d\left( t \right)$ and subtracted from the original signal, $r\left( t \right) = x\left( t \right) - g_{i} \left( t \right)$, where *i* refers to the *i*th IMF
7. We then begin from step 1, with the new signal *r*(*t*), and store $g_{i} \left( t \right)$ as an IMF.

The output of this algorithm is a series of IMFs and a final residual, *r*(*t*). There are two different stopping criteria for this algorithm: (1) based on definition of an IMF and (2) based on how many IMFs are produced. The definition of an IMF is related to the frequency span of the window of signal analysed. The original criteria used definitions based on narrowband frequency definitions in terms of the difference in number of extrema (maxima and minima) and zero crossings (that they must differ at most by 1). The number of IMFs produced depends on the methodology used to interpolate through the extrema. In conventional Hermite cubic spline methods, we are limited by three points.

Advantages of EMD

1. The EMD reduces the size or span of given signal by producing a collection of intrinsic mode functions (IMF).
2. Hilbert spectral analysis of IMFs result in empirical time–frequency, from which instantaneous frequency can be calculated easily.
3. Instead of unvarying amplitude and frequency in a simple harmonic component, an IMF can have changeable amplitude and frequency along the time axis.
4. No requirement of any kind of prior fixed basis functions.

### Hilbert transform

Instantaneous frequency (IF) is a frequency of the signal at particular instant of time *t*. In Fourier analysis, one complete oscillation of a sine or cosine function is needed to find out the local frequency (Huang et al.), but it could not make sense for non-stationary signals like EEG. There are dissimilar techniques to determine instantaneous frequency, but the preamble of the Hilbert transform with EMD is made easy and meaningful to discover IF.

In 1743, Leonard Euler (1707–1783) gave the formula ${\text{e}}^{jz} = \cos \left( z \right) + j\sin \left( z \right)$ is called Euler formula. After 150 years, Kennelly and P. Steinmetz used this formula to symbolize the complex notation of harmonic wave forms, i.e.${\text{e}}^{j\omega t} = \cos \left( {\omega t} \right) + j\sin \left( {\omega t} \right)$. In the commencement of the twentieth century, Hilbert demonstrated that the sin(*ωt*) function is the Hilbert transform of cos(*ωt*), and this gave the ± $\frac{\pi }{2}$ phase-shift operator which is an essential and basic property of the Hilbert transform. A real function *f*(*t*) and its Hilbert transform $\tilde{f}$(*t*) are correlated to each other in such a manner that they jointly make a strong analytic signal, and such signal can be represented by amplitude and a phase. The derivative of the phase can be identified as the instantaneous frequency (IF) of the signal at that instant of time. Function and its Hilbert transform have the same energy, and hence, the energy can be used to measure the calculated accuracy of the approximated Hilbert transform. The Hilbert transform in the time domain is characterized as a convolution between the Hilbert transformer $\frac{1}{{\left( {\pi t} \right)}}$ and capacity *f*(*t*). The Hilbert transform $\tilde{f}$(*t*) of a function *f*(*t*) for all time *t* is defined as follows:

4 $$\tilde{f}(t) = \frac{1}{\pi }P\int\limits_{ - \infty }^{ + \infty } {\frac{f\left( \tau \right)}{t - \tau }{\text{d}}\tau } .$$

when the integral exists. Here the P in front of the integral is known as Cauchy principal value.

Hilbert transform is used to produce an analytic signal. For a given signal *f*(*t*), and its Hilbert transform $\tilde{f}$(*t*), the analytical signal $f_{A}$(*t*) is given as follows:

5 $$f_{A} \left( t \right) = f\left( t \right) + j\tilde{f}\left( t \right)$$

The analytical signal is a complex signal that can be articulated in exponential notation:

6 $$f_{A} \left( t \right) = A\left( t \right){\text{e}}^{j\varPsi \left( t \right)}$$

where *A*(*t*) is the instantaneous amplitude (envelope), *ψ*(*t*) is the instantaneous phase.

Given the phase, we can calculate the instantaneous frequency:

7 $$I\left( t \right) = \frac{1}{2\pi }\psi (t){\text{d}}t.$$

IMF is a capacity or function with a similar number of extrema (minima and maxima) and zero intersections or crossing points, where envelopes are symmetric concerning zero. Therefore, definition of IMF guarantees a well-behaved Hilbert transform of the IMF. Hilbert spectral analysis (HSA), i.e. examination of each IMF’s instantaneous frequency as functions of time results in a frequency–time distribution of signal amplitude or energy, which allows the identification of localized features. The reason of choosing these papers is to motivate the researchers to do some investigation in this domain because in many cases the methods based on this domain are performing very well as compared to popular wavelet domain. Combination of features from different domain especially (IMFs + Frequency) is producing very dominating results. In this section, we have presented eight papers which make the readers familiar with famous time–frequency domain called EMD and a well-known and very useful transformation called Hilbert transform. All eight papers have different core ideas and approaches to detect seizures from EEG signals. Relationships among a variety of techniques have also been demonstrated. Results of EMD-based techniques are compared to wavelet and Fourier domain techniques and are better in many cases which motivate the researchers to carry out more investigation in EMD domain. Papers also help the readers to find the appropriate classifier and number of levels of IMFs.

Eftekhar et al. [37] Well-known time–frequency techniques like spectrograms and wavelet analysis have some issues like: both require some a priori knowledge of the signal and the assumption of linearity. Eftekhar et al. apply a new time–frequency technique called Hilbert-Huang technique or empirical time–frequency technique in seizure detection using EEG and ECG signal, and it is a combination of two famous methodologies of signal processing like Hilbert and Huang transform (Hilbert–Huang). It was initially proposed by Huang et al. (1996, 1998, 1999, 2003, 2009, 2012, 2013). They investigated this empirical time–frequency technique on EEG signal data (Freiburg [38]) and ECG signal data (CHB-MIT). After comparing the results with other existing time–frequency techniques, they believe that the study and the understanding of their methods is imperative and complimentary to existing time–frequency methodologies. Therefore, their results motivated the researchers to do more investigation by applying their proposed method for seizure detection.

Tafreshi et al. [39] In this paper, they used means of the absolute values of the IMF’s Hilbert transform as feature. They also compared their approach with another approach where feature extraction is done with wavelet transform. Algorithm used self-organizing map (SOM) neural networks and multilayer perceptron (MLP) classifiers for classification, and they showed that MLP are better than SOM networks. Results are tested on Freiburg database, where data is taken from 5 patients using 128 channels with 256-Hz samples. For each of the patients, there are datasets called “ictal” and seizures “inter-ictal”. They found window size of 1500 samples is optimum. In their findings, they revealed that the EMD approach is performing better than the wavelet approach, and they also mentioned that four empirical modes are enough to get better results. The MLP networks are superior in performance with 90.69% accuracy to the SOM networks having 87.28% accuracy for same four empirical modes.

Orosco et al. [40] In the proposed algorithm, energies of IMFs are used as discerning or discriminating features to differentiate seizure and non-seizure activities in an EEG signals at hand. In this approach, they compared the IMF energies with certain thresholds. The performance of the proposed algorithm is evaluated and tested on 9 patient records taken from Freiburg database which is invasive in nature. They used 9 patients and six channels, i.e. 3 focal electrodes and 3 extrafocal electrodes. The records are divided into segments 1 h long, and there are a total of 90 segments per channel, out of which 51 segments are non-seizure and 39 are epileptic seizures. The preprocessing of the data is done by notch filter, and then, EEG signals were band pass-filtered with a second-order, bidirectional Butterworth filter with a bandwidth of 0.5–60 Hz. They found sensitivity of 56.41% and specificity of 75.86%, respectively. It is single-channel and threshold-based method of seizure detection. No classifier is used here, and results are very poor as compared to the method proposed in [39].

Guarnizo and Delgado [41] In this paper, features used are amplitude, the average or instantaneous frequency for all EMD components, and also higher-order statistics such as the skewness and kurtosis in addition to Shannon’s entropy have been selected as features. The relevant features are selected with the help of mutual information (MI) which is the measure of relevance and redundancy among features. The classifier used here is linear Baye’s classifier, and a fivefold cross-validation is performed for different clinical cases. For classification, they integrated 4 IMFs and the residue of the EMD process. Different sets of features are tested with different group of datasets (Bonn database), and they found that combination of all features with different group of datasets have more accuracy. Algorithm achieved an average accuracy of 98%. It shows that up to 4 IMFs are sufficient, and results are good as compared to techniques proposed in [40, 41].

Sabrina et al. [42] The proposed technique for seizure detection is based on unsupervised learning (clustering techniques). Proposed algorithm used fast potential-based hierarchical agglomerative (PHA) clustering technique and empirical mode decomposition (EMD) process to distinguish a seizure from non-seizure activity. Distinguished features such as Euclidian, Bhattacharya and kolomogorov distances were computed between the IMFs and given as input for the PHA cluster algorithm. The performance of the proposed method is evaluated and tested on CHB-MIT, and they achieved promising results with an overall accuracy of 98.84%.

Dattaprasad et al. [43] In this paper, the EEG signal features are extracted using empirical mode decomposition (EMD) and artificial neural network (ANN) is used for classification and in the second step, classification to distinguish the signal class as seizure or non-seizure activities in EEG epochs in hand. Here, the signal is decomposed into intrinsic mode functions (IMFs) for the extraction of instantaneous frequencies; then, Hilbert transform is applied on each obtained IMFs by which feature coefficients are produced which tells the instantaneous frequency details. The performance of the proposed algorithm is tested on Bonn database and obtained an accuracy of 96%.

Alam and Bhuiyan [44] Here combined statistical and chaotic features like kurtosis, skewness, largest Lyapunov exponent, variance, approximate entropy, and correlation dimension from the first 4 IMFs components of EEG signals are used. Here an IMF is segmented into 16 blocks using a rectangular window of length 256. For each window, three chaotic features (LLE, CD, ApEn) and three statistical features (variance, skewness, kurtosis) are calculated. They used artificial neural network classifiers (ANN) for classification. The results are tested on Bonn database and algorithm achieved a sensitivity, specificity, and accuracy for (D,E) set using IMF3 and IMF4 of 100, 100, and 100%, respectively. They also showed that this method is superior as compared to other time–frequency algorithms like Liang et al., Tzallas, etc., in terms of computational complexity.

Bajaj and Pachori [45] They proposed an EMD-based seizure detection method to detect focal temporal lobe epilepsy. Algorithm used Hilbert transformation of IMFs which were obtained by an EMD process. Epileptic seizures are then detected based on the instantaneous area estimated from the trace of analytic IMFs of EEG signals. They decompose the signal up to IMF3 and found that IMF2 is sufficient to detect the onset seizures. The local mean of EMD can be used as a statistical feature for seizure detection. The performance of this algorithm was evaluated on Freiburg database. The sensitivity, specificity and error rate are of 90, 89.31, and 24.25%, respectively. It is a patient-specific algorithm.

In summary, we say that combination of EMD with Hilbert transform is performing exceptionally well as compared to other time–frequency domains. Up to four IMFs are sufficient to detect the onset seizure. Various classifiers are investigated, and in many cases they found artificial neural network is performing well. Unsupervised classifiers are also applied and tested, and it is totally a new approach in seizure detection.

*Observations* EMD is time frequency and adaptive in nature transformation. IMFs itself can be used as features because these components distinguish seizure and non-seizure portions very nicely. After analysing the above-mentioned methods, we can conclude that if EMD and wavelet transformations are combined, i.e. hybrid approach, this may give a new and better transformation in signal processing. But EMD still requires lot of investigations for its efficient use in signal processing.

## Section 4

### Rational transform or free parameter domain

This is also a time–frequency domain which is based on rational functions. It is adaptive in nature, i.e. basis functions are not fixed unlike Fourier and wavelet transforms. This method of feature extraction is already used in control theory and system to control the behaviour and identification of the system. The application of rational transform in seizure detection is totally new area. The coefficients of the rational transform decay very fast as compared to above-mentioned time–frequency domains. It is a free parameter-based technique where optimal basis are identified by using some optimization algorithms like particle swarm optimization (PSO) [46] or its variations. PSO is a population-based stochastic optimization algorithm developed by Dr. Eberhart and Dr. Kennedy in 1995, after inspiration from the social behaviour of bird flocking or fish schooling. The scenario is a group of birds are randomly searching for food, and there is only one piece of food in the area being searched. Initially, birds do not know where the food exactly is but they know how far the food is in each iteration. So the problem is to find the best strategy to find the food. The effective strategy is to follow the bird which is nearest to the food. PSO is initialized with a group of random solutions and then searches for optimal one by updating generations. PSO learned from the scenario, and it is used to solve the optimization problems. For more details about PSO, please refer the reference. Once the system is identified, rational transform can be applied. System parameters, i.e. poles itself can be used as features. The problem with the technique is that it requires complex computations to find the optimal set of basis or parameters from large number of free parameters. A parameter (pole) with multiplicities can be used as basis.

### Rational function system and orthogonality

Here we will discuss briefly about rational functions ant their orthonormality and orthogonality. Let ${\mathbb{C}}$ represent the set of complex numbers, ${\mathbb{D}}:\, = \left\{ {z \, \upepsilon \, {\mathbb{C}}:\left| z \right| < 1} \right\}$ the open unit disc, ℕ: = {1,2,3…} the set of natural numbers and ${\mathbb{T}}: \, = \{ z\, \upepsilon \, {\mathbb{C}}:|z| = 1\}$ the unit circle. Then the basic rational functions (RF) are defined as follows:

8 $$r_{a,k} \left( z \right) = \frac{1}{{\left( {1 - \bar{a}z} \right)^{k} }},(a\, \upepsilon \, {\mathbb{D}},z\, \upepsilon \, \bar{D},k\, \upepsilon \, {\mathbb{N}})$$

The parameter “*a”* is referred to as the inverse pole (because $\frac{1}{{\bar{a}}}$ is a pole), *k* is the order of the basic function. Using a terminology similar to the trigonometric case, the value *k* = 1 corresponds to the fundamental tone and *k* > 1 the overtones. Let us denote the proper rational functions that are analytic on the closed unit disc by *R*. Then, it can be shown that $\Re = {\text{span}}\left\{ {r_{ a,k } :a\, \upepsilon \, {\mathbb{D}}, \, k\, \upepsilon \, {\mathbb{N}}} \right\}$, i.e. any function *f* ϵ $\Re$ can be written as

9 $$f = \mathop \sum \limits_{j = 0}^{n} c_{j} r_{{ a_{j} k_{j} }} ,\left( {a_{j} \, \upepsilon \, {\mathbb{D}},k_{j} \, \upepsilon \, {\mathbb{N}}} \right)$$

The basic rational functions in Eq. (1) are linearly independent, but do not form an orthogonal basis, so it is difficult to compute the $c_{j}$ coefficients in Eq. (2). This problem can be solved by using the Gram–Schmidt orthogonalization procedure. The corresponding rational function system is the so-called Malmquist–Takenaka (MT) system. A handy property of the MT system is that the elements can be explicitly expressed as Blaschke products. Namely, taking a sequence of inverse poles $a_{0} , \ldots ,a_{n} \, \upepsilon \, {\mathbb{D}}$.

For a given $n\, \upepsilon \, {\mathbb{N}}$, *the MT system can be written as:*

$$\varPhi_{ k(z)} = \frac{{\sqrt {1 - \left| {a_{n } } \right|^{2} } }}{{\left( {1 - \bar{a}_{k} z} \right)}}\mathop \prod \limits_{j = 0}^{k - 1} B_{{a_{j} \left( z \right) }} ,$$

where 0 ≤ *k* ≤ *n* where $B_{{a_{j} \left( z \right) }}$ is called Blaschke function defined by:

$$B_{{a_{j} \left( z \right) }} = \frac{z - a}{{1 - \bar{a}z}},\quad \left( {z \, \upepsilon \, {\mathbb{C}} \bigg\backslash \left\{ {\frac{1}{{\bar{a}}}} \right\}} \right)$$

Although we have an orthonormal set of functions, the time localization property of the basic rational form has been lost. Fortunately, biorthogonal rational functions (BRF) cure this problem by keeping the orthogonality while avoiding the drawbacks of the MT system. This type of biorthogonal systems can be defined by taking *n* + 1 different poles $a_{0} , \ldots ,a_{n}$ with multiplicities $m_{0} , \ldots ,m_{n}$, and the corresponding modified rational base functions (MRF)

$$\varphi_{ k,i\left( z \right) } = \frac{{ z^{i - 1} }}{{\left( {1 - \bar{a}_{k} z} \right)^{i} }}\quad \left( {k = 0, \ldots n,\quad i = 1,m_{k} } \right)$$

We note that, the system of $r_{{ {{a}},{{k }}}}$ and $\varphi_{{ {{a}},{{k}}}}$ span the same subspaces of *R* for *a *≠ 0. For the definition of a biorthogonal system, the following functions are needed:

$$\varOmega_{\ell n} \left( z \right) = \frac{1}{{ \left( {1 - \bar{a}_{\ell } z} \right)^{{m_{\ell } }} }}\mathop \prod \limits_{i = 0,i \ne \ell }^{n} \left( {\frac{{\left( {z - a_{\ell } } \right)}}{{\left( {1 - \bar{a}_{i} z} \right)}}} \right)^{{m_{i} }}$$

$$\omega_{\ell n} \left( z \right)\frac{{\varOmega_{\ell n} \left( {a_{\ell } } \right)}}{{\varOmega_{\ell n} \left( z \right)}},\;{\text{Where}}\left( {0 \le \ell \le n} \right).$$

By theorem 1 in [47] the (BRF) functions

$$\varPsi_{l,j} \left( z \right) = \frac{{\varOmega_{\ell n} \left( z \right) \left( {z - a_{\ell } } \right)^{j - 1} }}{{\varOmega_{\ell n} \left( {a_{\ell } } \right)}}\mathop \sum \limits_{s = 0}^{{m_{\ell } - j}} \frac{{ \omega_{\ell n}^{\left( s \right)} \left( {a_{\ell } } \right)}}{{s^{!} }}\left( {z - a_{\ell } } \right)^{s}$$

($0 \le \ell \le \, n, \, 1 \le \, j \, \le m_{\ell }$) are biorthogonal to $\varphi_{{ {{a}},{{k}}}}$ with respect to the scalar product:

$$\begin{aligned} & \frac{1}{2\pi }\mathop \int \limits_{ - \pi }^{\pi } F\left( {{\text{e}}^{it} } \right)\bar{G}\left( {{\text{e}}^{it} } \right){\text{d}}t\quad \left( {F,G\, \upepsilon \, H^{2} ({\mathbb{T}})} \right) \\ & \left\langle {\varPsi_{k\ell } ,\varphi_{ks} } \right\rangle = \delta_{k\ell } \delta_{rs } \\ \end{aligned}$$

(1 ≤ $r$ ≤ *n*, 1 ≤ ${{m}}_{\ell } , 1 \le {{s}} \le {{m}}_{{k }}$, 0 $\le$ *k*, $\ell \le {{n}})$ and $\delta_{{ij }}$ is well-known Kronecker delta symbol. We note that the previously defined rational function systems are complete in the Hardy space $H^{2} \left( {\mathbb{H}} \right)$; if and only if the so-called Blaschke condition is satisfied [48]:

$$\mathop \sum \limits_{n = 0}^{\infty } \left( {1 - \left| {a_{n } } \right|} \right) = \infty$$

Then for a finite set of poles, the MT and the biorthogonal systems span the *n* and *N* dimensional subspaces of $H^{2} \left( {\mathbb{H}} \right)$, respectively:

$$p_{\phi }^{n} f = \mathop \sum \limits_{k = 0}^{n - 1} \left\langle {f,\varPhi_{k} } \right\rangle \varPhi_{k}$$

$$p_{\varPsi }^{n} f = \mathop \sum \limits_{k = 0}^{n - 1} \left\langle {f,\varPhi_{ki} } \right\rangle \varPhi_{ki}$$

where $$N = m_{0} + m_{1} + \cdots + m_{n - 1}$$.

We note that the MT and the biorthogonal systems *Φ* and *Ψ* with the basic rational functions are referred as the rational orthogonal basis (ROB) in the literature. The construction of these generalized orthogonal bases was introduced by Heuberger et al. [48]. Till now we have seen many transformations and decomposition techniques to extract the features from the EEG signal. In this section, we are giving the overview of the various papers which are based on rational function system. The idea of feature extraction is totally new in the field of EEG seizure detection. The advantages of rational function systems over the other well-known transformation methods are: (a) *flexibility*, which means, not only the coefficients but also the system (orthogonal system) itself can be changed (system can be personalized or adapted to the EEG biomedical signal), (b) the coefficients generated from the rational system give a very compact representation of the signal, and hence, they can be used as features to detect the seizures, (c) the elementary waves are contained or localized in time, and hence, the basis functions can hold time–frequency information, (d) rational system gives a simple analytic representation of the original signal, (e) only a small number of arithmetic operations are required to recover the signal.

Samiee et al. [49] Proposed a new method of feature extraction in time–frequency domain called MT rational DSTFT which relies on rational function, and it is adaptive in nature. Their method proposed a sparse representation of the signal while the components remain orthogonal. They investigated that the best window and coefficients size are 256 samples (1.5 s) and first 32 coefficients of the proposed transform. Authors applied stochastic hyperbolic particle swarm optimization (PSO) algorithm to find the optimal position of the pole of each EEG epoch which gives the compact *t*–*f* representation of the proposed system. For seizure detection, features used are absolute mean value, absolute median value, absolute standard deviation, absolute maximum value, absolute minimum value of the coefficients. The performance of the proposed method is evaluated on **Bonn database** and showed that the algorithm has more accuracy (in terms of sensitivity keeping specificity fixed) than other *t*–*f* transforms like DSTFT and 13 Cohen’s transforms with the same number of nonzero coefficients and achieved an accuracy of 99.8 and 99.3 for the combination of E–A and E–B datasets, respectively. They also showed that the inverse rational DSTFT has smaller MSEs as compared to classical inverse DSTFT. But reliability of the algorithm needs to be checked for long EEG data. They investigated various algorithms and found feed-forward MLP as optimal classifier. Their proposed future work is to improve this proposed algorithm by using multidimensional (MD) PSO to determine the optimal number of unique poles so that rational DSTFT can be used in multichannel long-term epileptic seizure detection algorithm.

Samiee et al. [50] Here, they concentrated and solved the problem of off-line supervised detection of epileptic seizures in long-term EEG recording. To achieve the goal, they developed a new feature extraction method, which is based on the sparse rational decomposition and the local Gabor binary patterns (LGBP). In the proposed algorithm, they decompose the EEG channels or signals into 8 sparse rational components by using a set of most favourable coefficients. Next, a modified 1D LGBP operator is applied, which is followed by down-sampling of the data. The efficiency or success of the proposed method of feature extraction is tested by means of dissimilar classifiers. The proposed algorithm is tested on CHB-MIT scalp EEG database from PhysioNet using EEG recording of 163 h. Their proposed technique performs better over dedicated and well-known techniques (wavelet, STFT, etc.) by showing an overall sensitivity and specificity of 70.4 and 99.1%, respectively. Their algorithm detects commencement of seizures with an average overall sensitivity of 91.13% and false alarms per hour rate of 0.35.

Fridli et al. [51] Used rational function system for the analysis of the ECG signals. Their technique has many advantages over the previously used generalized techniques like wavelet transform. Their system is very specific for ECG signals and shape of the individual term correspond to the natural shape of the ECG signals. The system is flexible, i.e. the coefficients and the system itself can be optimized even from heartbeats to heartbeats. The system is simple and less number of calculations are required. They used the terminology fundamental tone for $r_{ a,1 }$ and overtone for $r_{ a,k }$ if *k *>1. Linear combinations of basic functions having the same pole will be called elementary functions or elementary waves:

10 $$E_{ a } \left( z \right) = \mathop \sum \limits_{k = 1}^{n} c_{ k } r_{ a ,k } \left( z \right) = \mathop \sum \limits_{k = 1}^{n} \frac{1}{{\left( {1 - \bar{a}z} \right)^{k} }}$$

The expression “elementary wave” is justified by the fact that such a function can be well localized in a proper neighbourhood of the pole. Although the elementary waves are defined on the entire set of complex numbers but interested mainly in the real parts of their restrictions on the torus ${\mathbb{T}} = \left\{ {z\, \upepsilon \, {\mathbb{C}}:\left| z \right| = 1} \right\}$. The result can be naturally associated with the real–real function:

$$\left[ { - \pi ,} \right){ \ni }t \to {\text{e}}^{it} \to \text{Re} \left( {E_{ a } \left( {{\text{e}}^{it} } \right)} \right)$$

The above-mentioned idea demonstrates their idea behind the proposed approach. Their idea is: Let us first choose 3 inverse poles, $a_{ i }$= $\left| {a_{ i } } \right|{\text{e}}^{{i\alpha_{ i } }}$ within the unit disc. They are visualized in Fig. 1a.
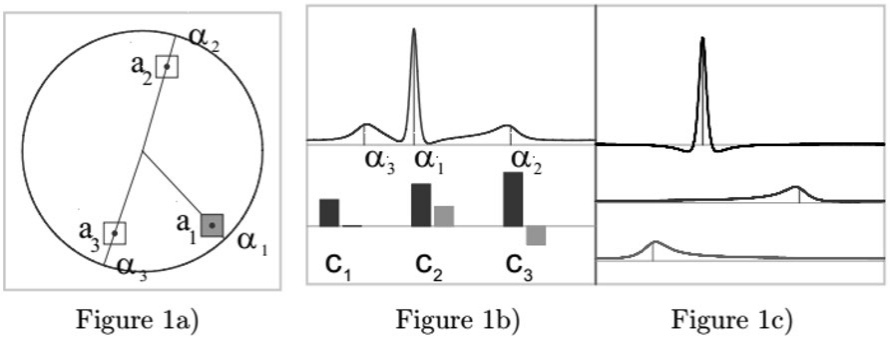


Then they take the linear combination of two fundamental tones $r_{{a_{2 } ,1 }}$, $r_{{a_{3 } ,1 }}$ and of one overtone of second degree $r_{{a_{2 } ,2 }}$. In the bottom of Fig. 1b, there are the complex coefficients and the top part is the graph of the resulting rational function. The top entry in Fig. 1c is the overtone $c_{1 } r_{{a_{1 } ,2 }}$, the middle one is the fundamental tone $c_{2 } r_{{a_{2} ,1}}$, and the bottom one is $c_{3 } r_{{a_{3 } ,1}} .$ It is visible that the shape of the rational function is similar to that of an ECG signal, the segmentation corresponds to the terms, and the $\alpha_{ i }$ parameters of the inverse poles carry time information.

*Observations* This type of domain is totally new in seizure detection where basis of the transformations are adaptive in nature. Rational function basis are well suited while doing the analysis of the ECG signals. Such types of systems are flexible, simple, and less number of calculations is required and can be used in real-time applications.

## Conclusion

The aim of this study is to help the researchers to get familiar with state-of-the-art techniques for seizure detection and give them the valid research directions in the field of seizure detection. In this paper, some hints about the amount of work that has already been done for various databases and self-recorded data are given. Various methods from various domains used to process long-term and short-term EEG signals are discussed. We have seen that almost all researchers have done the EEG analysis by similar four steps: (1) raw signal processing, (2) feature extraction, (3) best feature selection, and (4) two-class classification. In most of the papers, authors combine features from different domains to construct the feature vector and in many cases their methods are performing exceptionally well as compared to features from single domain. Five-level decomposition using wavelet transform is good choice for extracting efficient features. We have seen that EMD-based methods outperformed than wavelet-based methods in many cases, and it is good choice to use IMFs for feature extraction but still requires further investigations. Rational transform is totally new method for feature extraction in seizure detection. The comparison and summary of various techniques is summarized in Table 1.

**Table 1:** Summary of various seizure detection algorithms

Authors	Year	Features/transforms used/channels	Classifiers	Window size	Dataset	Performance metrics (%)
Runarsson et al. (T)	2005	Amplitude and time difference/single channel	SVM	Frames are not fixed	Self-recorded	Sensitivity: 90%
Alejandro et al. (T)	2017	Energy/multichannel	Genetic algorithm/threshold	NA	CHB-MIT	False rate 0.39 per 24 h in average
Turky et al. (T)	2016	Histograms	Threshold value	10 s	CHB-MIT	97.14 sens., 98.58 spec.
Mursalin et al. (T)	2017	Mean, mode, etc./wavelets	Random forest (RF)	NA	Bonn dataset	Avg acc. 98.45
Michael	2016	FFT coff.	Random forest (RF)	1 s	Self-rec. by compi.	NA
Supratak et al.	2014	NA	NA	NA	CHB-MIT	Sensitivity: 100%, false positive rate: 7.98/h, detection latency: 6.87%
Yoo et al. (T)	2013	Energy/multichannel	SVM	2 s	CHB-MIT	Accuracy: 84.4%, classification
Ahamad et al.	2014	NA	NA	NA	CHB-MIT	Sensitivity: 98.5%, detection latency: 1.76 s
Kiranyaz et al.	2014	NA	NA	NA	CHB-MIT	Sensitivity: 89%
Dalton et al. (T)	2012	Signature of seizure/single channel	Template matching	12–25 s	Dataset of 21 seizure	Sensitivity: 91%, specificity: 84%, battery lifetime: 10.5 h
Rana et al. (F)	2012	Phase slope Index/Fourier/multichannel	None	10–60 s	EEG and ECoG time series, BW: 0.5–100 Hz	Accuracy: 100%
Khamis et al. (F)	2013	Frequency moment signatures/Fourier/single channel	Powell’s direction set method	32 s	12 patients with six data records (R1 to R6)	Sensitivity: 91%, false positives per hour: 0.02
Acharya et al. (F)	2012	Four entropies/single channel	SVM, FSC, PNN, KNN, NBC, DT, and GMM	23.6 s	Channel Self-recorded data	Average accuracy: 98.1%
Zhou et al. (W)	2012	Lacunarity and fluctuation index on wavelet scales/multichannel Wavelet	Bayesian linear discriminant analysis (BLDA) classifier	4 s without overlapping	Freiburg database of 21 patients	Sensitivity: 96.25%, false detection rate: 0.13/h, mean delay time: 13.8 s
Hasan. et al. (w)	2016	Mean, maximum, minimum, standard deviation, and average power of absolute values of wavelet and Hilbert transform coefficients	K-nearest neighbourhood (kNN)		Bonn database	Accuracy: the wavelet 100 and 96% for the A–E and B–E datasets, respectively, the Hilbert: transform 100 and 100% for the A–E and B–E datasets, respectively
Liu et al. (W)	2012	Relative energy, relative amplitude, coefficient of variation, fluctuation index/multichannel/wavelet	SVM	4 s	Dataset of 509 h from 21 epileptic patients	Sensitivity: 94.46%, specificity: 95.26%, false detection rate: 0.58/h
Panda et al. (W)	2010	Energy, entropy, standard deviation/single channel/wavelet	SVM	0.5 s	500 epochs of EEG data from five different brain activities (100 signals per epoch)	Accuracy: 91.2%
Guangyi et al.	2017	Amplitude of coefficients/wavelet/Fourier	KNN		Bonn database	Classification rates (100%)
Khan et al. (W)	2012	Energy and normalized coefficient of variation/single/wavelet	Simple LDA classifier	25 s	Self-recorded	Sensitivity: 83%, specificity: 100%, accuracy: 92%, overall precision: 87%
Rezvan et al.	2017	Maximum, minimum, average and standard deviation/single/wavelet	MLP Neural network	26.3 s	Bonn database	confusion matrix: accuracy, 98.33
Wang et al.		Approximate entropy/single/wavelet	Neyman–Pearson criteria and SVM	70–200 ms	Changhai Hospital database (scalp EEG)	Detection accuracy: 98%, false detection rate: 6%
Zainuddin et al.	2012	Maximum, minimum, standard deviation of absolute wavelet coefficient/single/wavelet	WNN	23.6 s	Bonn database	Sensitivity: 98%, accuracy: 98%
Niknazar et al.	2013	Time delay, embedding dimension/single/wavelet	ECOG	23.6 s	Bonn database	Accuracy: 98.67%
Daou and Labeau	2014	SPIHT codes/single/wavelet	No classifier	1 s		Accuracy: 90%
Shoaib et al.	2014	Wavelet energy/multichannel/wavelet	SVM	2 s	MIT database (scalp EEG	Sensitivity: 91–96%, latency: 4.7–5.3 s, false alarm rate: 0.17–0.3/h
Zandi et al.	2010	Combined seizure index/single/wavelet	No classifier	10–40 s	EEG recordings from 14 patients approximately 75.8 h with 63 seizures	Sensitivity: 90.5%, false detection rate: 0.51 h-1, median detection delay: 7 s
Tafreshi et al. (E)	2008	Mean of the absolute of each IMF, wavelet feature/single/EMD	Neural Network	4–6 s	Five patients of Freiburg database (scalp and iEEG)	Accuracy: 95%
Orosco et al.	2009	Energies of the IMFs/single/EMD	No classifier	I h	90 EEG segments acquired for nine patients	Sensitivity: 56%, specificity: 75%, positive predictive value: 61%, negative predictive value: 72%
Guarnizo and Delgado	2010	Instantaneous frequency, amplitude of each EMD component, skewness, kurtosis, Shannon’s entropy/single/EMD	Linear Bayes classifier	Whole signal	Five groups with 100 single-channel registers sampled at 173.61 Hz	Accuracy: 98%
Alam and Bhuiyan	2011,2013	Skewness, kurtosis, variance, largest Lyapunov exponent, correlation dimension, approximate entropy from intrinsic mode/single/EMD	ANN	23.6 s	Bonn database	Accuracy: 100%
Bajaj and Pachori	2014	Modified central tendency measure/single/EMD	No classifier	15 s	Freiburg database for 21 patients (scalp and iEEG)	Sensitivity: 90%, specificity: 89.31%, error detection rate: 24.25%
Sabrina et al.	2016	Euclidian, Bhattacharya and kolomogorov (IMFs)	PHA(unsupervised)	2 s	CHB-MIT	Accuracy of 98.84%
Dattaprasad et al.	2017	Coefficients of Hilbert(IMF)/EMD/Hilbert	ANN	23.6 s	Bonn database	Accuracy of 96%
Kaveh et al.	2014	Absolute mean, median, standard deviation, maximum and minimum value of the coefficients/rational STFT	MLP	1.5 s	Bonn database	Accuracy: E–A: 99.8 E–B: 99.3, etc.
Kaveth and Peter		LGBP/1 D LBP/multiple channel	SVM/RF/Log-reg	1	CHB-MIT	Sensitivity of 70.4% and the overall specificity of 99.1%

## Future research directions

After going through a large number of papers, we found that lot of work is done in time and frequency domain features and we feel that there is a need to find such features which measure the gap of EEG patterns. There is a need to develop and implement high-speed and accurate algorithms in various hardware devices which are used to detect seizures. Furthermore, it is quite challenging to integrate the techniques with various datasets and therefore requires lot of efforts and investigation in this direction too and to concentrate to develop new methodologies to extract features from the signals. The use of rational functional system to extract the features from EEG signals to detect seizures is completely new area but still lot of investigations are required in this field. Researchers may be required to try free parameter-based orthogonal basis (i.e. basis changing segments to segments) because P. Kovacs et al. tried the same and got promising results with less computations. The EEG data is increasing day by day; the researchers need to develop the algorithms which are producing promising results on large data. Channel selection is also a quite challenging task, and some efforts are still needed in this area too. To find the exact start and end of the seizure period is challenging. One may try to use spectral analysis, because it is a very powerful feature of extraction techniques like wavelet. Parameter optimization may also require innovative way. Many researchers used single channel approach for seizure detection but multichannel techniques are performing exceptionally well, so there is scope to carry out research in this area. Other transformations like piecewise linear transformations need to be tested on discrete type biomedical signals.
